# Nonsurgical Aesthetic Improvement of the Neck: A Multimodal, Experience‐Based Algorithmic Approach

**DOI:** 10.1111/jocd.71012

**Published:** 2026-07-03

**Authors:** Dagne Pupo Ricardo, Arash Jalali, Kyu‐Ho Yi

**Affiliations:** ^1^ Dagne Pupo Clinic Mallorca Spain; ^2^ One Clinic MD Vancouver Canada; ^3^ You and I Clinic Seoul Republic of Korea

**Keywords:** botulinum toxin, cervicomental angle, high‐intensity focused ultrasound, hyaluronic acid filler, multimodal treatment, neck rejuvenation, platysma, polynucleotides, radiofrequency microneedling, submental fat

## Abstract

**Background:**

Aesthetic aging of the neck is usually multifactorial, reflecting variable contributions from submental and cervical adiposity, dermal and septal laxity, platysmal activity, skeletal deficiency, and loss of lower‐face support. Isolated nonsurgical treatment may therefore underperform when the dominant anatomical driver is not correctly identified.

**Objective:**

This narrative review presents a practical, multimodal, algorithm‐based framework for nonsurgical neck rejuvenation, integrating current evidence with clinical experience across commonly used treatment modalities.

**Methods:**

Relevant literature on lipolytic therapies, energy‐based devices, structural fillers, collagen stimulators, intradermal treatments, and neuromodulators was selectively reviewed and synthesized with clinical experience to develop an experience‐informed treatment framework.

**Results:**

Patients may be broadly categorized into three dominant but overlapping phenotypes: volume excess, skin laxity, and structural deficiency. Volume‐dominant patients may benefit from initial volume reduction before tightening or structural refinement, whereas laxity‐dominant patients may require energy‐based remodeling and dermal‐quality improvement. Patients with structural deficiency may benefit from chin or mandibular support to improve cervicomental balance. Adjunctive intradermal therapies and neuromodulators can be incorporated according to residual skin‐quality changes and muscular contribution.

**Conclusion:**

Nonsurgical neck rejuvenation is best approached as a staged, multimodal, lower‐face‐inclusive process rather than treatment of an isolated cervical concern. The proposed framework is intended as a practical decision‐support tool for individualized treatment planning, not as a validated or prescriptive treatment protocol.

## 1 Introduction

The neck occupies a disproportionately important role in perceived facial age and harmony. Patients may tolerate modest periorbital or lower‐face aging, yet remain highly dissatisfied with blunting of the cervicomental angle, submental fullness, platysmal banding, horizontal neck lines, or the loss of a clean mandibular‐neck transition. Classic surgical literature has long emphasized that youthful neck contour depends on a favorable relationship between skin quality, subcutaneous fat, deeper support structures, the platysma, and osseous projection of the chin and mandible [1]. In practice, however, patients increasingly seek office‐based options that can improve the neck without formal cervicofacial surgery. The expanding range of minimally invasive and nonsurgical techniques has made this request more realistic than in previous decades, but it has also introduced increasing complexity in clinical decision‐making. Rather than a lack of treatment options, the current challenge lies in selecting and sequencing appropriate modalities for a given neck phenotype [2, 3, 4, 5, 6]. The modern nonsurgical armamentarium includes injectable lipolytics, cryolipolysis, endolaser‐assisted adipose reduction, radiofrequency microneedling, ultrasound‐based tightening, hyaluronic acid fillers, calcium hydroxyapatite, poly‐L‐lactic acid, polycaprolactone‐based biostimulatory products, neuromodulators, skin boosters, and polynucleotides [2, 3, 4, 5, 6, 7, 8, 9, 10, 11, 12, 13, 14, 15, 16, 17, 18, 19, 20, 21, 22, 23, 24, 25, 26, 27, 28, 29, 30]. Each modality affects a different anatomical layer or biological pathway. Lipolytic therapies are most relevant when submental or jowl‐associated volume predominates; energy‐based devices address laxity and collagen remodeling; fillers and biostimulators restore contour and support; neuromodulators reduce unfavorable muscular pull; and intradermal regenerative products improve the quality of the skin envelope. The therapeutic challenge is therefore largely diagnostic and architectural. In many cases, the apparent clinical presentation may reflect overlapping anatomical contributors. For example, a neck that appears heavy may in part be influenced by microgenia or mandibular retrusion, while apparent laxity may coexist with submental fullness. Because the neck and lower third of the face are visually continuous, isolated treatment of one zone may risk accentuating rather than correcting imbalance [3, 4].

This narrative review presents a clinically oriented, algorithm‐based framework for nonsurgical neck rejuvenation by integrating commonly used modalities into a structured, stepwise approach. The intent is not to define a rigid or prescriptive protocol, but to synthesize available evidence alongside clinical experience and translate these into a practical decision‐support tool.

For clarity, patients can be broadly grouped into three dominant but often overlapping categories: those requiring volume reduction, those in whom skin laxity predominates, and those with structural deficiency of the chin or mandible. These categories are intended to guide initial treatment prioritization rather than represent fixed or mutually exclusive classifications. The overarching principle is that successful neck rejuvenation depends on identifying the dominant anatomical driver while recognizing the aesthetic interdependence of the neck and lower third of the face.

## 2 Scope and Review Approach

This paper is structured as a narrative review with an algorithm‐oriented clinical focus. The aim is not to perform a formal systematic review or meta‐analysis, but to synthesize available literature and integrate it with clinical experience to support practical decision‐making in nonsurgical neck rejuvenation.

Relevant studies, including randomized trials, prospective studies, systematic reviews, and expert consensus publications, were selectively considered with emphasis on anatomical understanding, patient selection, treatment mechanisms, sequencing strategies, and safety considerations. Given the relative scarcity of high‐level, neck‐specific evidence for several modalities, selected literature from lower‐face and facial aesthetic treatments was also included where it provides clinically applicable insights.

The proposed algorithm should therefore be interpreted as a conceptual and experience‐informed framework rather than a validated or prescriptive treatment pathway. Variability in study design, treatment parameters, and outcome measures across the literature limits direct comparison and generalization, and clinical judgment remains essential in adapting the framework to individual patients.

## 3 Anatomical and Aesthetic Basis of Neck Aging

Neck aging is anatomically layered. At the most superficial level, the skin progressively loses elasticity because of intrinsic aging, cumulative photodamage, and changes in dermal collagen and elastin. This produces surface laxity, rhytids, and textural decline. Beneath the skin, subcutaneous fat may accumulate in the submental region, laterally at the jowls, or diffusely in the upper cervical field. In some patients, a deeper component of fullness also develops, making the cervicomental angle appear obtuse even when superficial skin quality is acceptable [1, 2, 3, 4]. The platysma adds a dynamic component. With time, platysmal tone and banding can become more visible, contributing both vertical neck bands and a downward vector on the mandibular border and lower face [3, 26, 27, 28]. Skeletal support is equally important. Microgenia, retrognathia, or age‐related bony resorption can reduce the visual tension of the neck by diminishing anterior chin projection and weakening the jawline as a stable superior frame [1, 4, 16].

This explains why the neck cannot be fully understood in isolation. The cervicomental angle is affected by the position of the chin, the contour of the mandibular line, the presence of jowls, and the balance between lower‐face elevators and depressors. When the jawline is poorly projected, the neck may appear fuller even if true cervical adiposity is modest. When jowling is prominent, a neck‐focused treatment that ignores the mandibular border may produce an incomplete or disharmonious result. Chiu et al. [4] emphasized that lower‐face and neck assessment should be unified rather than compartmentalized, because aesthetic improvement depends on transitions between zones rather than on a single point correction. Hu et al. [3] similarly highlighted that neck aesthetics are strongly influenced by platysmal patterning and mandibular contour, particularly when neuromodulators are used as part of a lower‐face lifting strategy.

A further consequence of layered aging is that a single intervention rarely addresses the whole problem. For example, fat reduction in a patient with poor skin recoil may initially worsen the impression of laxity by deflating a region without improving the envelope. Conversely, collagen stimulation without reducing excessive submental fat may tighten the surface but leave the cervicomental contour unchanged. Structural jawline or chin filler in a patient with untreated jowls or heavy submental tissue may create a sharper mandibular boundary above an unchanged heavy neck, making the contrast more obvious rather than more youthful. This is why a layered, sequence‐based strategy is more rational than modality‐based enthusiasm. The question should not be which treatment is fashionable, but which tissue is dominant, which sequence best reveals the residual deficit, and which combination is most likely to improve harmony [2, 31, 32].

## 4 Lipolytic and Volume‐Reduction Strategies

When excess volume is the primary deforming force, reduction should usually precede tightening or structural augmentation. This principle is straightforward: a heavy submental compartment or pronounced jowl can obscure the cervicomental angle and mandibular contour to such an extent that later interventions cannot be properly judged until the excess adipose burden has been reduced. The literature supports several nonsurgical approaches for localized fat reduction, including cryolipolysis, injectable lipolysis, deoxycholic acid, endolaser‐assisted contouring, and more recently, enzymatic lipolysis [7, 8, 9, 10, 11].

Cryolipolysis has been reviewed as a nonsurgical option for submental fat reduction, with the attraction of non‐injectable treatment and relatively low downtime [7]. It can be useful in patients with localized submental fullness and adequate baseline skin quality, particularly when avoidance of needles is a major preference. Injection lipolysis has a longer and more heterogeneous history, with variable formulations and techniques described across the literature [8]. Although broad conclusions are limited by product variability, the core concept remains that chemically induced adipocytolysis can improve localized adipose fullness when patient selection and dosing are appropriate.

Among injectable lipolytic approaches, deoxycholic acid has the strongest formal evidence base. A systematic review and meta‐analysis of randomized controlled trials found that deoxycholic acid significantly improved submental contour compared with placebo across multiple efficacy endpoints [9]. This evidence has been important because it provides a level of rigor that many neck modalities still lack. At the same time, clinical practice shows that swelling, tenderness, induration, and transient contour irregularity can limit enthusiasm unless patients are appropriately counseled. Deoxycholic acid is most useful when adiposity is localized and the skin envelope is reasonably resilient. It is less satisfying when laxity predominates or when the patient expects a surgical‐quality change from injections alone.

Endolaser‐based fat reduction has become increasingly relevant because it may combine focal adipose reduction with thermal tissue contraction. Although treatment parameters, fiber placement, and experience vary considerably, the appeal is obvious: a single intervention can reduce local fat while also initiating dermal and fibroseptal remodeling. Park et al. [10] recently reviewed lipolytic agents for submental reduction and emphasized that treatment choice should be individualized according to adipose distribution, skin laxity, and the need for simultaneous tissue contraction. Similarly, Jabbour et al. [11] reported prospective data on an enzymatic mixture combining lipase, collagenase, and hyaluronidase for moderate to severe submental fat, suggesting that enzyme‐based approaches may broaden the nonsurgical armamentarium in carefully selected patients.

**FIGURE 1:**
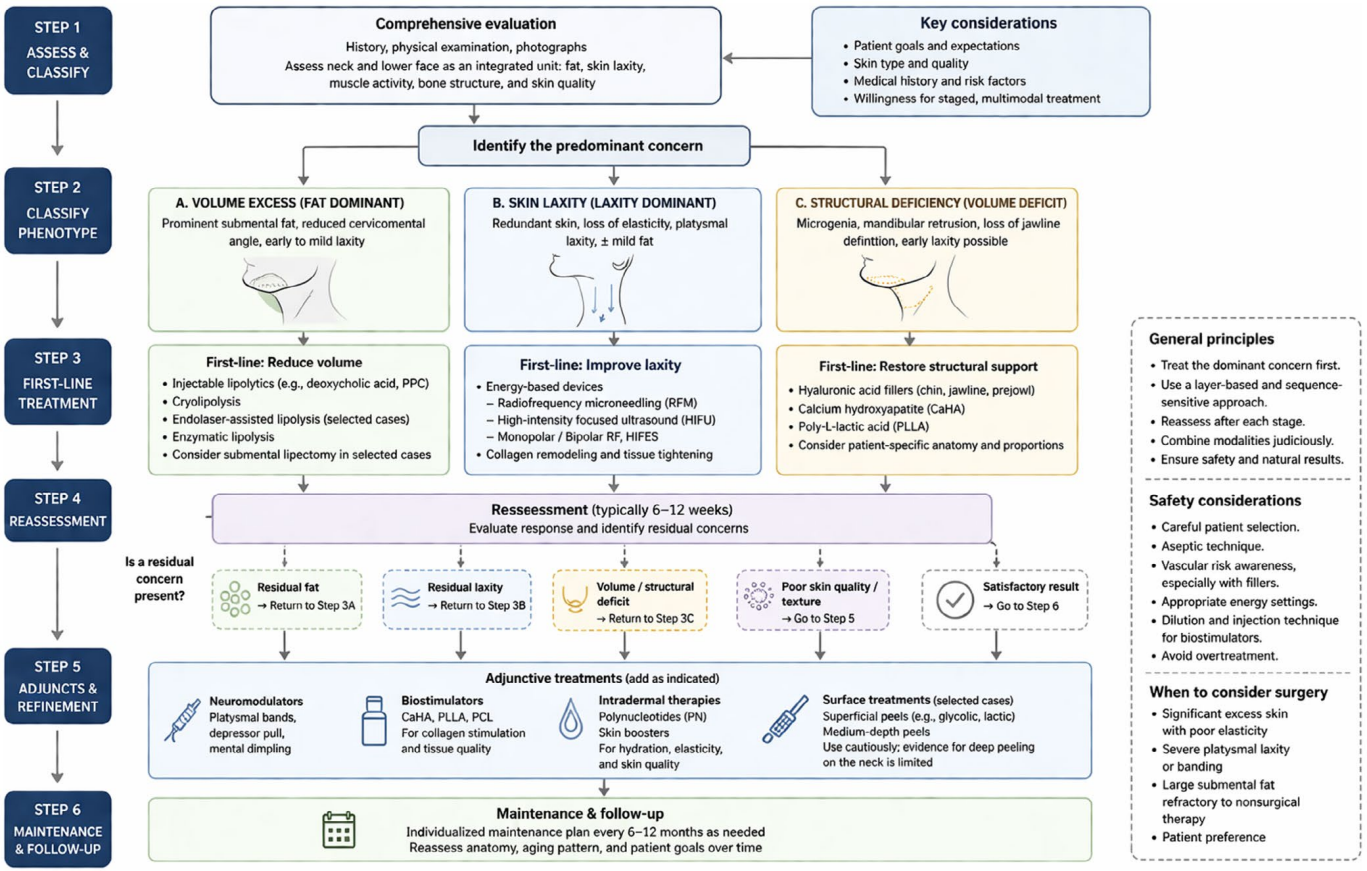
Conceptual, experience‐informed framework for nonsurgical neck rejuvenation. The framework begins with comprehensive assessment and classification into three dominant phenotypes (volume excess, skin laxity, and structural deficiency), followed by phenotype‐guided treatment planning and reassessment. It is intended as a clinical decision‐support tool for individualized treatment planning rather than a validated treatment algorithm or protocol. CaHA, calcium hydroxyapatite; EBD, energy‐based devices; HIFES, high‐intensity focused electrical stimulation; HIFU, high‐intensity focused ultrasound; PCC, phosphatidylcholine; PCL, polycaprolactone; PLLA, poly‐L‐lactic acid; PN, polynucleotides; RF, radiofrequency.

What matters clinically is not only the efficacy of a lipolytic modality in isolation, but its place in the overall sequence. Within this framework, patients with volume‐dominant features are often initially managed with volume reduction, as this can establish a clearer baseline for subsequent assessment. However, treatment sequencing remains flexible and is guided by individual anatomy and response to prior interventions (Figure 1). Once the fat burden is improved, the residual phenotype becomes clearer. Some patients require no further contour intervention beyond maintenance. Others show unmasked skin laxity and are better served by RFM or HIFU. Still others benefit from subsequent neuromodulation or filler because the improved submental field reveals persistent platysmal pull or poor mandibular definition.

## 5 Energy‐Based Devices for Skin Tightening and Remodeling

Energy‐based devices occupy a central role in nonsurgical neck rejuvenation because they target the structural basis of laxity rather than merely filling or masking its consequences. Radiofrequency microneedling and high‐intensity focused ultrasound are the most widely discussed modalities, although combined radiofrequency systems and radiofrequency with high‐intensity facial electromagnetic stimulation have also been used in selected protocols [12, 13, 14, 15]. Their value lies in collagen remodeling, dermal tightening, and, in some cases, subdermal contraction. Importantly, these effects are most useful when the patient has mild to moderate laxity and realistic expectations about the magnitude and tempo of change.

A systematic review of radiofrequency for face and neck rejuvenation concluded that radiofrequency‐based approaches can improve skin laxity and textural quality with an acceptable safety profile, although heterogeneity in devices, treatment parameters, and outcome measures remains substantial [12]. High‐intensity focused ultrasound has a different mechanism, producing focal zones of thermal coagulation in deeper layers and thereby stimulating tightening over time. Kumar et al. [13] reviewed prospective and experimental studies of HIFU for facial and body contouring and found consistent support for its use as a nonsurgical tightening tool, again with the caveat that energy settings, target depths, and patient selection substantially influence outcomes.

The neck is particularly well suited to an energy‐based strategy when there is visible skin laxity after weight fluctuation, age‐related thinning, or prior fat reduction. However, the clinical endpoint is not simply tighter skin. The real goal is restoration of smoother transitions from jawline to cervicomental angle and from neck to décolleté, with improved resistance to dynamic folding. Gold et al. [14] demonstrated that combined bipolar radiofrequency and infrared treatment can improve wrinkles and overall skin tone and texture, which, although not neck‐specific, supports the concept that thermal‐based remodeling can enhance the superficial aesthetic envelope. In neck practice, this matters because even a well‐defined jawline can appear old if the overlying cervical skin remains crepey, banded, or irregular.

Energy‐based devices are also important because they interact favorably with other treatments. Tam et al. [15] systematically reviewed combination approaches using biostimulators with botulinum toxin, fillers, and energy‐based devices and concluded that multimodal protocols can offer synergistic aesthetic benefit when properly sequenced. In the neck, this synergy is intuitive. Fat reduction may create a more favorable contour platform for RFM or HIFU. Thermal remodeling may in turn improve the tissue bed before or after biostimulatory injection. A patient with both adiposity and laxity may therefore be better treated with staged lipolysis followed by energy‐based tightening than with either modality alone.

## 6 Structural Fillers and Volume Replacement

The concept of volume replacement in neck rejuvenation initially seems paradoxical to patients who feel that the neck looks full or heavy. In reality, however, structural volume loss in the chin and mandible is one of the main reasons the neck looks less defined. If the anterior chin is underprojected or the mandibular line lacks angularity, the cervicomental angle becomes visually blunted even before true cervical aging is severe. For that reason, group C in the algorithm includes patients whose main need is structural support rather than direct neck reduction (Figure 1).

Hyaluronic acid fillers are the most familiar tools for restoring chin and jawline support. Ogilvie et al. [16] reported safe and effective chin and jaw restoration with VYC‐25L, demonstrating how structural augmentation can strengthen lower‐face contour in ways that indirectly enhance neck definition. In practical terms, a stronger chin creates better anterior tension on the neck profile, whereas a cleaner mandibular line improves the perceived border between face and neck. This does not mean that filler should replace treatment of true submental fullness. Rather, it means that neck rejuvenation should be interpreted through the framework of support and proportion, not only tissue subtraction.

Fillers also have a role in treating static horizontal neck rhytids and selected superficial contour irregularities. Siperstein et al. [17] reported one‐year data on hyaluronic acid filler for static horizontal neck rhytids, supporting the idea that carefully placed filler can improve line‐dominant cervical aging in selected patients. A broader systematic review of facial hyaluronic acid filler durability is relevant because it reminds clinicians that longevity depends on product properties, injection plane, movement, and treatment site [18]. Neck tissue is highly mobile, so durability and palpability can differ from midface experience. Product choice, plane, and amount therefore matter more than brand familiarity alone.

Biostimulatory fillers and collagen inducers expand the concept of volume replacement beyond simple gel implantation. Hyperdiluted calcium hydroxyapatite has been widely discussed as a face and body biostimulatory agent, with consensus recommendations describing its utility for improving tissue quality and mild laxity when appropriately diluted and layered [19]. Poly‐L‐lactic acid has a longer history as a collagen stimulator, and international consensus literature continues to refine its role in body and structural rejuvenation [20]. Polycaprolactone‐based devices and combination strategies, including sutures and HA, have also been explored in the face and neck [21]. From a neck perspective, the importance of these agents lies in their capacity to treat laxity and support together. They are not interchangeable with hyaluronic acid, but they are particularly useful when the goal is progressive tissue remodeling rather than immediate edge definition.

A key principle is that volumization for neck improvement should be structural and strategic, not indiscriminate. Overfilling the prejowl sulcus or mandibular border in a heavy neck can create a bulky or masculinized appearance. Similarly, superficial use of high G‐prime products in mobile cervical skin can produce visibility or stiffness. The best nonsurgical results usually arise when the practitioner identifies whether the missing element is chin projection, mandibular continuity, dermal support, or a combination thereof, and then selects product and plane accordingly. In this sense, volume replacement is not the opposite of reduction; it is the complementary act of rebuilding an adequate frame after the heavier or lax elements have been managed.

## 7 Intradermal Bioremodeling, Skin Boosters, and Polynucleotides

A recurrent limitation of purely structural or contractile therapies is that they do not fully address the quality of the skin envelope. Neck aging often includes fine wrinkling, poor hydration, surface irregularity, and a crepey texture that remains visible even after the cervicomental contour improves. This is the clinical niche of intradermal treatments such as skin boosters and polynucleotides. These therapies are not substitutes for volume reduction, tightening, or structural support; instead, they enhance the dermal substrate in ways that can improve the overall quality of the result [22, 23, 24, 25].

The consensus report on polynucleotides in aesthetic medicine emphasized their regenerative positioning and their role in improving tissue quality, hydration, and recovery [22]. Rho et al. [23] similarly reviewed injectable skin boosters as tools for aging skin rejuvenation, describing their relevance to texture, elasticity, and dermal hydration. Although much of the evidence has focused on facial skin rather than the neck specifically, the underlying rationale is applicable: a neck with improved contour but poor dermal quality often still looks aged. Niforos et al. [24] demonstrated that VYC‐12 injectable gel improved facial skin topography, supporting the broader principle that intradermal hyaluronic formulations can enhance superficial skin characteristics.

In the neck, intradermal bioremodeling is particularly useful after primary contour work has been completed. For example, a patient in group A may benefit from polynucleotides or a skin booster after lipolysis and RFM once inflammation has settled and the residual issue is mainly skin texture or mild surface laxity. Likewise, a patient in group B may undergo serial RFM or HIFU with adjunctive intradermal treatment to improve hydration, resilience, and subjective skin quality. Cheng et al. [25] reviewed regenerative skincare technologies derived from human fibroblasts, including growth factors and exosomes, and highlighted both the promise and the immaturity of this space. For current clinical purposes, however, polynucleotides and skin boosters are more established than topical regenerative narratives and fit more naturally into a staged neck protocol.

## 8 Neuromodulators in Neck and Lower‐Face Harmonization

Neuromodulators have become increasingly important in neck and lower‐face rejuvenation because they treat a key but sometimes underappreciated driver of aging: unfavorable muscular pull. The platysma contributes not only to visible neck bands but also to downward tension on the jawline and lower face. Similarly, interaction among the masseter, depressor anguli oris, platysma, and mentalis affects mandibular contour and lower‐face balance. When these vectors are excessive, structural treatments may be undermined by persistent dynamic descent [3, 26, 27, 28].

Kaufman‐Janette and Trindade de Almeida [26] described lifting with neuromodulators as an increasingly refined concept based on selective weakening of depressor patterns rather than global paralysis. In the neck, this logic has now been supported by formal trials. Fabi et al. [27] reported Phase 3 evidence showing improvement of platysma prominence with onabotulinumtoxinA in a randomized, double‐blind, placebo‐controlled study. Shridharani et al. [28] likewise demonstrated improved neck and jawline aesthetics by minimizing platysma contraction effects in a Phase 3 randomized, placebo‐controlled trial. These publications are important because they move neck neuromodulation from an expert technique toward a more evidence‐based standard.

In practice, neuromodulators are rarely used as the sole treatment for an aged neck, but they can substantially improve the final outcome when incorporated thoughtfully. A patient with mild laxity and prominent banding may respond well to toxin plus RFM or a biostimulator. A patient undergoing jawline filler may benefit from prior or concurrent platysmal relaxation to reduce downward vector competition. Goodman et al. [29] showed improved patient satisfaction after combined filler and onabotulinumtoxinA treatment in the periorbital region, illustrating the broader principle that multimodal treatment can improve both appearance and perceived naturalness. Although not neck‐specific, the logic applies to lower‐face and neck practice as well. Dosing also matters. Philipp‐Dormston et al. [30] emphasized the importance of dose–response behavior with onabotulinumtoxinA, which is particularly relevant in the neck because underdosing may yield little change, whereas poorly distributed overdosing can compromise expression or function. A recent review of facial and cervical botulinum toxin techniques further underscored the value of viewing the face and neck as a unified muscular field rather than a set of disconnected injection zones [33].

## 9 Practical Multimodal Algorithm

The proposed framework is centered on three dominant treatment phenotypes, as illustrated in Figure 1. The framework is structured as a stepwise process that incorporates initial assessment, phenotype classification, first‐line intervention, and iterative reassessment before escalation or refinement. Group A includes patients in whom volume excess is the primary clinical driver, Group B includes those in whom skin laxity and tissue quality predominate, and Group C includes patients with structural deficiency of the chin or mandibular line. Although overlap among these patterns is common, this classification is intended to guide initial treatment prioritization rather than define rigid or exclusive categories. In clinical practice, many patients require staged or combined approaches, and the framework should be applied flexibly rather than as a fixed protocol.

The principal nonsurgical modalities used in this multimodal framework and their practical treatment characteristics are summarized in Table 1.

**TABLE 1:** Principal nonsurgical modalities for neck rejuvenation and their practical treatment characteristics.

Modality	Primary role	Typical sessions	Time to visible response	Downtime	Maintenance	Combination use
Lipolytic treatments	Localized submental/jowl volume reduction	1–5	3–8 months	4–15 days	8–12 months	Often first‐line when bulk dominates
Energy‐based devices	Skin tightening and collagen remodeling	1–5	30–60 days	0–7 days	4–12 months	Commonly combined with lipolytics or biostimulators
Hyaluronic acid fillers	Structural support and selected line correction	1–3	Immediate to 60 days	3–21 days	6–24 months	Useful for chin, jawline, and selected static neck lines
Collagen inducers	Biostimulation and tissue support	1–3	About 60 days	4–15 days	6–24 months	Often combined with EBDs or HA filler
Skin boosters	Hydration, texture, and superficial skin quality	1–3	About 30 days	0–7 days	3–12 months	Adjunctive rather than structural
Polynucleotides	Regenerative dermal support and tissue quality	3–5	About 90 days	0–7 days	6–12 months	Adjunctive, often used after contour correction
Neuromodulators	Reduction of platysmal pull and banding	1	4–15 days	Hours to 2 days	3–6 months	Can be staged before or after filler and devices

In group A, the primary objective is to reduce localized adiposity while preserving or improving the skin envelope. Lipolytic approaches are often considered as an initial step in appropriately selected patients, although treatment choice depends on anatomical characteristics and patient preference. Options may include deoxycholic acid, cryolipolysis, endolaser, or enzymatic lipolysis [7, 8, 9, 10, 11].

Following initial volume reduction, reassessment is important to identify residual concerns. Some patients may require no further contour intervention beyond maintenance, whereas others may demonstrate unmasked skin laxity that can be addressed with energy‐based devices. In selected cases, adjunctive neuromodulation or structural support may be considered if persistent platysmal activity or mandibular insufficiency contributes to the residual aesthetic imbalance.

In group B, the dominant concern is typically skin laxity and tissue quality rather than volume excess. These patients often present with mild to moderate laxity of the lower face and central neck, with associated textural changes or dynamic banding. Energy‐based devices such as radiofrequency microneedling or HIFU are commonly used as foundational treatments [12, 13].

However, because laxity reflects multiple structural factors, combination approaches may be considered. Biostimulatory treatments and intradermal therapies may be incorporated to improve dermal quality, while selective structural support, such as chin augmentation, may be appropriate in some patients to enhance the cervicomental transition.

In group C, the clinical emphasis shifts toward structural support. In some patients, apparent neck aging is influenced by reduced chin projection or mandibular definition rather than primary cervical pathology. In such cases, structural filler may be considered as a primary intervention, with or without adjunctive treatment of residual submental volume.

The aim is not to replace volume reduction or skin treatment, but to restore an adequate structural framework that can improve overall cervicomental balance. In appropriately selected patients, this approach may provide a more efficient pathway to aesthetic improvement compared with repeated surface‐based interventions alone.

Across all three groups, treatment sequencing remains an important consideration; however, the sequence described in this framework should be interpreted as a conceptual guide rather than a validated clinical pathway. When volume excess is present, it is often addressed early in treatment planning, whereas in volume‐neutral patients, energy‐based or biostimulatory approaches may be prioritized. Structural filler may be introduced once support needs are clearly defined, rather than applied indiscriminately at the outset.

Intradermal skin‐quality treatments are generally positioned as adjunctive interventions, often after primary contour correction. Neuromodulators may be incorporated at different stages depending on muscular contribution and treatment goals. The optimal sequence may vary between patients, but a staged approach with periodic reassessment is generally preferred, as each intervention may alter the perception of the remaining aesthetic deficit.

## 10 Illustrative Cases

**FIGURE 2:**
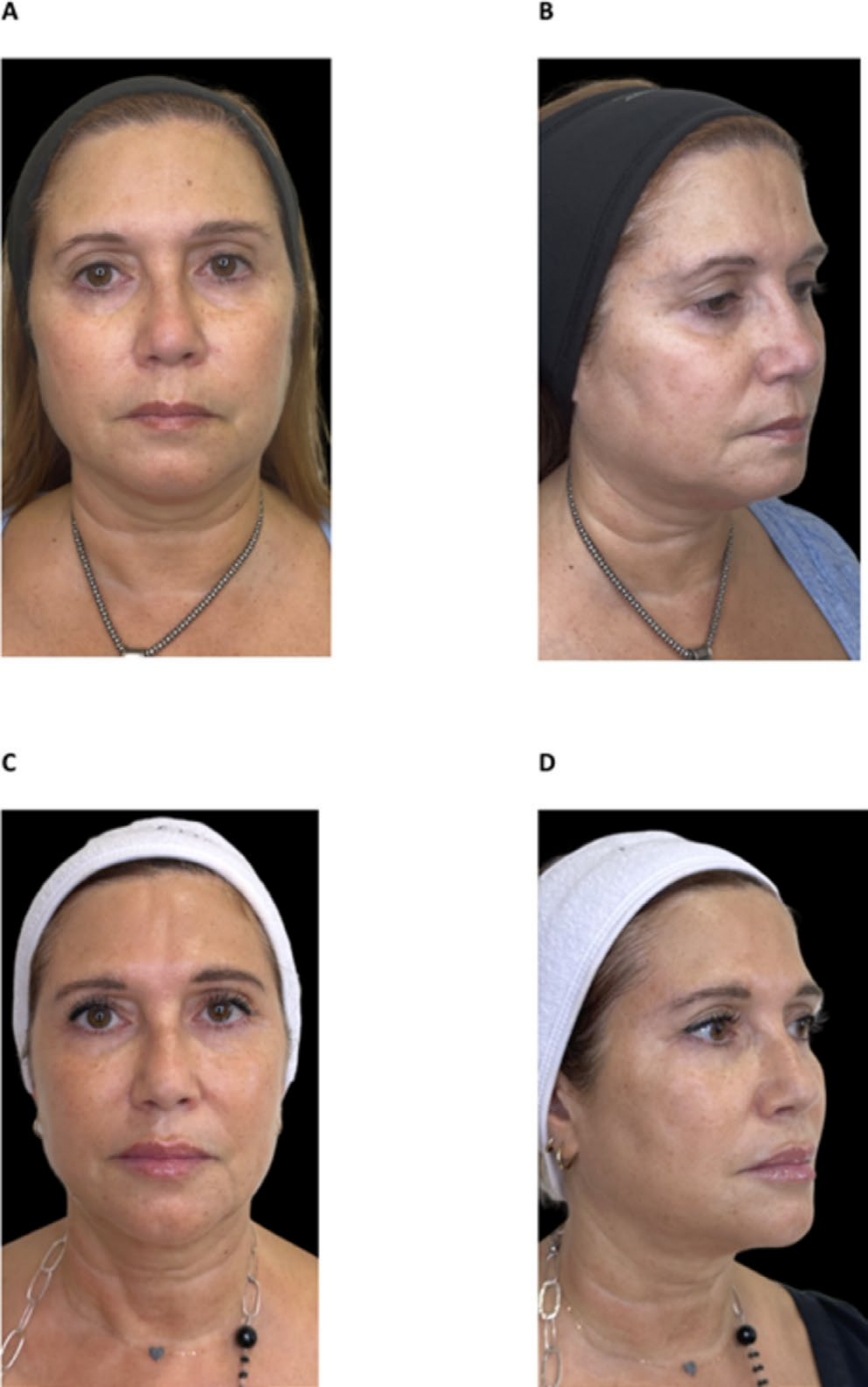
Representative group A case (volume reduction dominant): A 62‐year‐old woman shown before and after staged multimodal treatment involving fat reduction, remodeling, neuromodulation, and mandibular support. Panel A and B, before the treatment and Panel C and D are after the treatment.

The group A case shown in Figure 2 illustrates a volume‐dominant phenotype managed through staged multimodal treatment. The patient, a 62‐year‐old woman, underwent a combination of lipolytic therapy, energy‐based remodeling, biostimulation, neuromodulation, and subsequent mandibular support.

The 12‐month outcome demonstrates improvement in cervicomental contour; however, the result likely reflects the combined effect of both neck‐directed and lower‐face interventions rather than a single dominant modality. This case highlights the importance of staged treatment and reassessment in complex, multilayered presentations.

**FIGURE 3:**
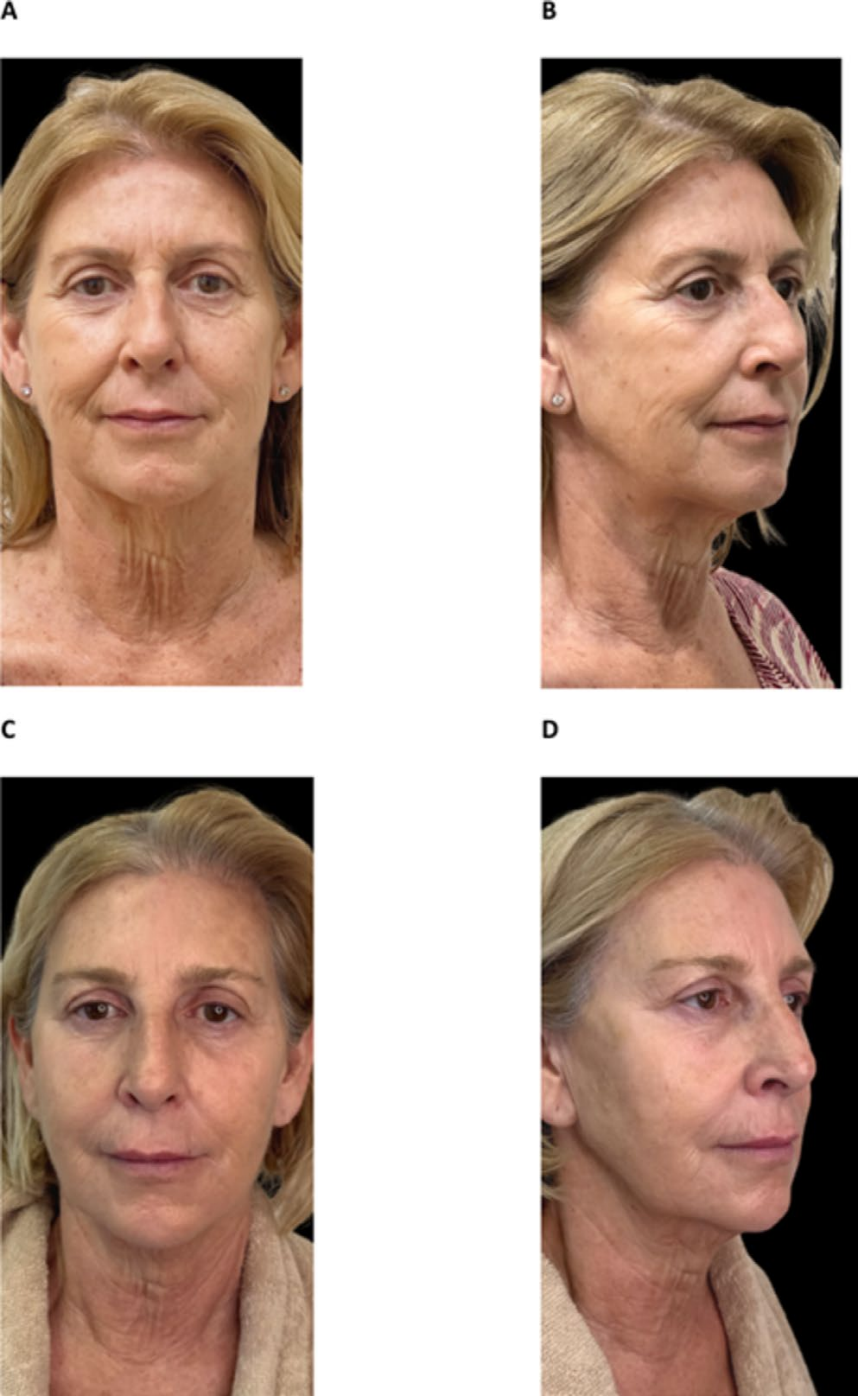
Representative group B case (skin tightening dominant): A 58‐year‐old woman shown before and after multimodal treatment with energy‐based remodeling, biostimulation, intradermal therapies, neuromodulation, and selective structural support. Panel A and B, before the treatment and Panel C and D are after the treatment.

The first group B case, shown in Figure 3, represents a laxity‐dominant presentation managed with repeated remodeling and adjunctive therapies. The treatment plan included energy‐based devices, biostimulatory agents, intradermal treatments, neuromodulation, and selective structural support.

The observed improvement reflects cumulative effects across multiple anatomical levels, including both the neck and lower face. As such, the result should be interpreted as a multimodal outcome rather than an isolated cervical correction.

**FIGURE 4:**
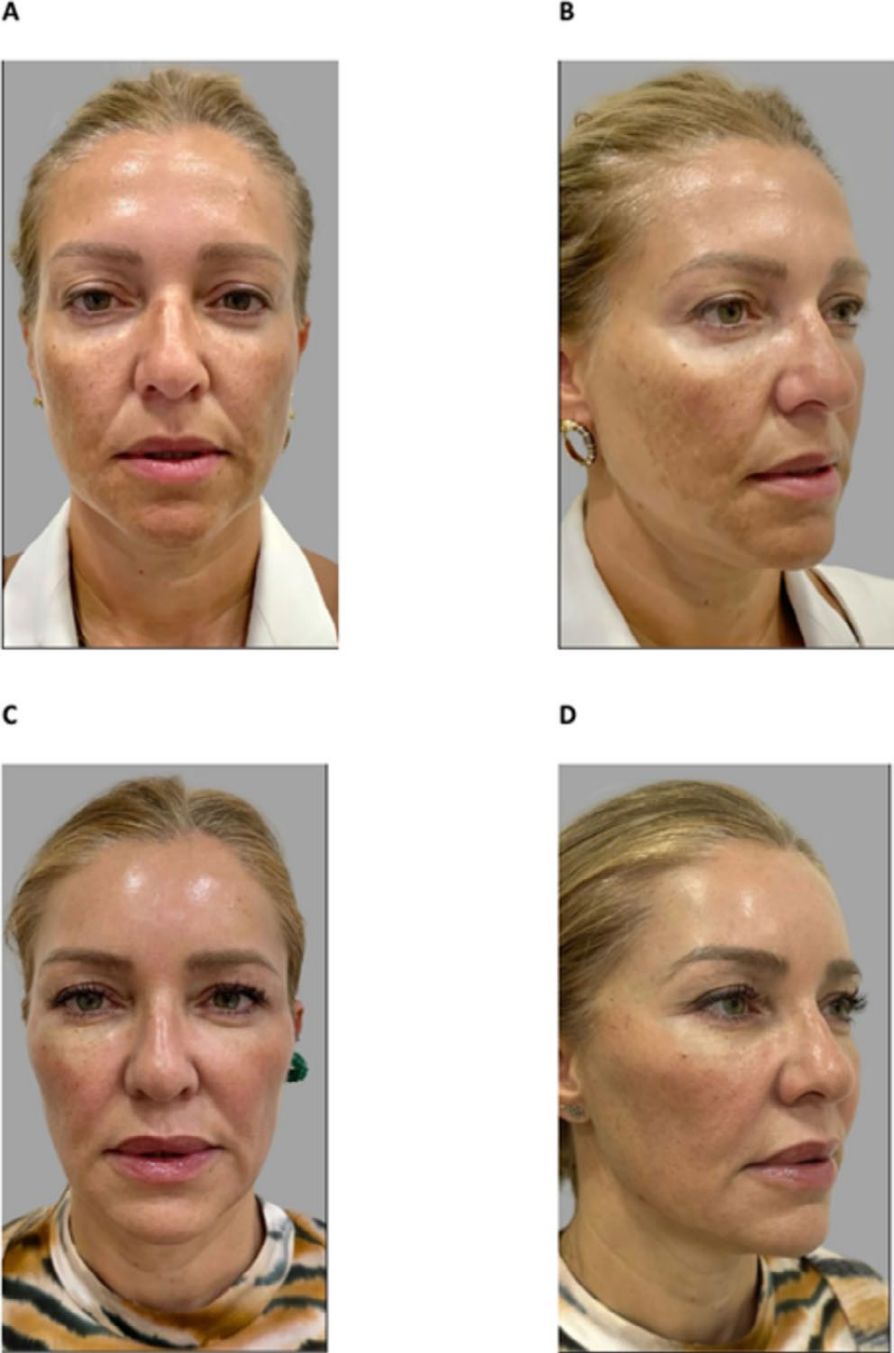
Representative group B case with longer follow‐up: A 46‐year‐old woman shown before and after extended multimodal tightening with HIFU, RF/HIFES, PLLA, repeated radiofrequency microneedling, endolaser, and neuromodulators. Panel A and B, before the treatment and Panel C and D are after the treatment.

The second group B case, shown in Figure 4, represents a younger but more structurally lax phenotype with longer follow‐up. This 46‐year‐old woman underwent HIFU to the mandibular line and neck, radiofrequency with HIFES to the submental region and midface, PLLA to the submental area and neck, repeated radiofrequency microneedling, endolaser to the jowl‐submental‐neck continuum, and later neuromodulators. The 3‐year comparison is informative because it shows how persistent maintenance and combination therapy can stabilize results in patients who are not ideal surgical candidates or who prefer a progressive nonsurgical pathway.

**FIGURE 5:**
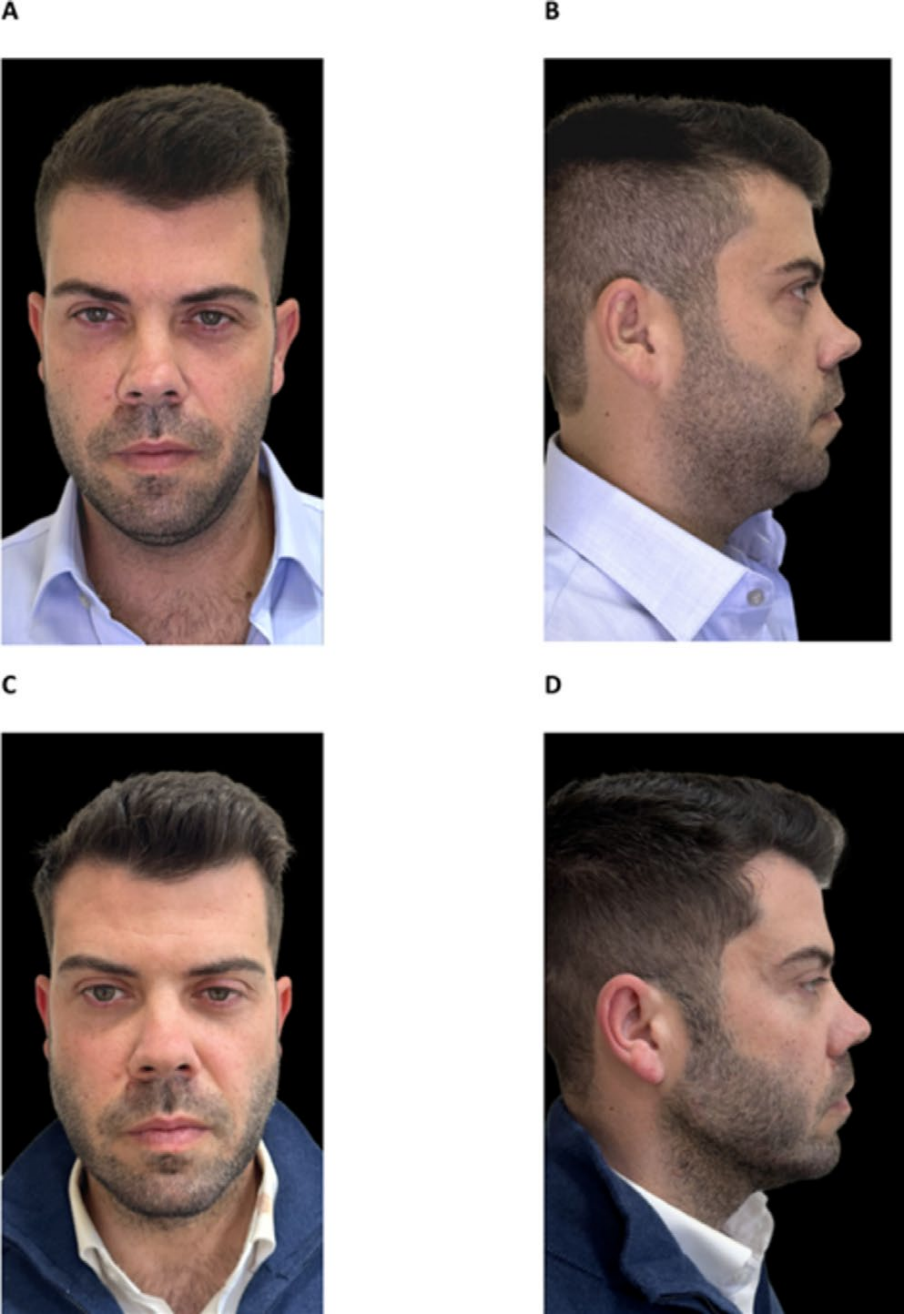
Representative group C case (structural deficiency dominant): A 38‐year‐old man shown before and after mandibular‐line HA filler with adjunctive submental treatment, demonstrating improved profile balance. Panel A and B, before the treatment and Panel C and D are after the treatment.

The group C case, shown in Figure 5, highlights the role of structural support in cervicomental aesthetics. The patient underwent mandibular‐line hyaluronic acid filler with adjunctive submental treatment.

The resulting improvement is likely driven primarily by enhanced mandibular definition and profile balance, rather than direct modification of cervical tissue alone. This case underscores the importance of recognizing structural deficiency as a contributing factor in selected patients.

## 11 Discussion

One of the key contributions of an algorithmic approach is to promote diagnostic discipline rather than oversimplification. In aesthetic practice, clinicians often become highly skilled with one or two favored modalities and then begin to see most problems through those tools. The neck resists that tendency because its visible aging is rarely monolayer. A neck that appears full may be adipose‐dominant, skeletal‐deficient, platysma‐dominant, laxity‐dominant, or mixed. A treatment algorithm forces the practitioner to identify the primary driver and to decide what should be treated first, what should be reassessed later, and what can be deferred. This reduces both overtreatment and mistreatment.

The algorithmic approach also reinforces the importance of treating the neck and lower third of the face as a continuous aesthetic unit. This is arguably the central conceptual point of the paper. Traditional treatment planning often separated the neck from the jawline, chin, and lower‐face musculature, yet patients do not see these areas as independent. They perceive profile, definition, and contour transitions. A modest increase in chin projection can transform the neck profile. Relaxation of platysmal pull can sharpen a jawline without directly adding volume. Reduction of jowl bulk can make the cervicomental angle appear cleaner even before the neck itself is treated. In this sense, successful neck rejuvenation is often an exercise in harmonization rather than direct cervical correction [3, 4, 16, 26, 27, 28].

A further advantage of the multimodal framework is safety. When interventions are sequenced logically, the amount of product or energy required at later stages is often reduced. For example, lipolysis before filler may lower the filler volume needed for mandibular definition. Energy‐based tightening after initial fat reduction may treat the residual problem more precisely than if applied through a heavy submental pad. Neuromodulators placed after structural contouring can be adjusted to the now‐visible vector pattern rather than injected empirically. These are not merely aesthetic refinements; they may reduce the likelihood of heaviness, irregularity, or patient dissatisfaction.

The case material reinforces this point. The illustrative cases shown in Figures 2, 3, 4, 5 demonstrate different entry points into a multimodal treatment strategy rather than serving as controlled evidence of efficacy.

The group A case demonstrates that a heavy lower face and neck may require fat reduction, remodeling, myomodulation, and later contour support. The group B cases show that laxity can be improved through repeated remodeling and biostimulation even when surgery is not pursued. The group C case emphasizes that some apparently cervical problems are actually problems of support and balance. Together, the figures illustrate that multimodal planning is not synonymous with maximal treatment. Rather, it is the selective layering of complementary modalities over time.

The current literature provides varying degrees of support for individual components of a multimodal treatment philosophy, but important limitations remain. Compared with the midface, lips, or upper face, the neck remains underrepresented in high‐quality randomized studies. Deoxycholic acid and onabotulinumtoxinA currently have stronger evidence than many other neck interventions, whereas RFM, HIFU, fillers, and biostimulatory protocols are supported by a mixture of systematic reviews, prospective studies, expert consensus, and clinical experience. Accordingly, the proposed framework should be regarded as an evidence‐informed conceptual model intended to assist clinical decision‐making rather than as a validated treatment algorithm.

Another important issue is patient communication. Multimodal neck treatment often requires multiple sessions, staged outcomes, and combination therapy. Patients accustomed to single‐visit filler correction in other facial regions may underestimate the time and sequencing needed for the neck. The review by Gregory et al. [31] on retention rates after multimodal facial rejuvenation is relevant here because it suggests that patients accept and continue multistep plans when the rationale is clear and the outcomes are meaningful. Similarly, the HARMONY study showed that multimodal facial aesthetic treatment can improve not only visible aging but also social confidence and psychological well‐being [32]. Although these studies are not neck‐specific, they support a broader observation familiar to experienced practitioners: patients tend to accept staged treatment when the plan is coherent and the relationship between modalities is explained.

The concept of adjunctive or holistic support deserves brief comment. Some practitioners incorporate nutritional optimization, collagen supplementation, or regenerative skincare into aesthetic care. Wicklund [34] reviewed how nutrition and supplements may influence aesthetic outcomes, which is a reasonable reminder that tissue quality does not arise in a vacuum. However, these measures should not be overstated. They may support general skin health, but they do not replace structural treatment of adiposity, platysmal pull, or skeletal deficiency. Likewise, regenerative technologies such as growth‐factor or exosome‐enriched topicals remain promising but variably evidenced [25]. In the context of a neck algorithm, they are adjuncts rather than pillars.

A final point concerns reproducibility. An algorithm becomes useful only if it is adaptable to real patients with overlapping phenotypes. Very few necks belong purely to one category. Some patients have both microgenia and submental fat. Others have undergone weight loss and present with simultaneous laxity, banding, and structural deficiency. The value of grouping patients into A, B, or C is therefore not absolute classification, but prioritization. It tells the clinician where to start. In many cases, the first step reveals the true need. This is one reason why algorithmic thinking is so powerful in aesthetic medicine: it allows reassessment to be built into the treatment plan rather than interpreted as failure.

The review has several limitations. It is narrative rather than systematic, and it depends partly on evidence that is modality‐specific rather than directly comparative. Some cited studies address facial or lower‐face outcomes more broadly than cervical outcomes alone. Several therapies remain off‐label in specific jurisdictions, and product behavior can vary substantially depending on technique, dilution, and tissue thickness. The case figures are illustrative rather than controlled data.

Nevertheless, the framework may serve as a practical tool for integrating available evidence and clinical experience into individualized treatment planning for nonsurgical neck rejuvenation.

## 12 Conclusion

Nonsurgical rejuvenation of the neck is most effectively approached as a multimodal, lower‐face‐inclusive process rather than as isolated treatment of a single visible concern. The framework presented here outlines three practical starting phenotypes—volume excess, skin laxity, and structural deficiency—which may guide initial treatment prioritization.

From these entry points, treatment can be sequenced and adapted using a combination of lipolytic therapies, energy‐based devices, structural fillers, collagen stimulators, intradermal treatments, and neuromodulators. The proposed approach emphasizes staged intervention and reassessment rather than fixed protocols. As the evidence base for neck‐specific nonsurgical treatments continues to evolve, such conceptual frameworks may help support more consistent, individualized, and balanced aesthetic outcomes in clinical practice.

## Author Contributions

**Dagne Pupo Ricardo:** conceptualization, writing – original draft, writing – review and editing, visualization, supervision. **Arash Jalali:** writing – original draft, writing – review and editing, visualization, supervision. **Kyu‐Ho Yi:** writing – original draft, writing – review and editing, visualization, supervision. All authors have reviewed and approved the article for submission.

## Funding

The authors have nothing to report.

## Disclosure

The authors have nothing to report.

## Ethics Statement

This study complied with the Declaration of Helsinki.

## Consent

Written informed consent was obtained from all patients for treatment and for the use of their clinical images for publication.

## Conflicts of Interest

The authors declare no conflicts of interest.

## Data Availability

Data sharing is not applicable to this article as no datasets were generated or analyzed during this study.
